# Institutional Notes and News

**Published:** 1910-07-30

**Authors:** 


### Institutional Notes and News.

**The Melbourne Hospitals.**

The Bank overdraft is now £4,000, and the State Treasurer has promised to "try" to increase the State grant of £10,000 by another £1,000 for next year. The Women's Hospital, for the rebuilding of which infirmary a wide appeal has been made to the public by bazaar and otherwise, proposes to add a third story to the new building for the accommodation of twelve septicæmia cases at a time. This will enable them to admit outside cases. The Fairfield Hospital for Infectious diseases is usually lacking in funds owing to the different modes of payment adopted by different municipalities; some contribute their quota, some pay 7s. 6d. a week for cases they send in, and others pay nothing at all. Under section 154 of the Health Act the defaulters are now to be compelled to take one of three courses: erect and maintain their own hospitals; combine and enter into an agreement with some hospital to treat their cases, or make use of the existing institution. The proposed consumptive hospital, to be erected at Cheltenham, a seaside resort, is being fiercely objected to by the inhabitants as a menace to their health and destructive of the value of their properties. A still more forcible argument is that the Australian sea coast, owing to rapid changes of temperature, is not suited to these cases. The hospital should be built inland on a height.

**Leicester Infirmary.**

The half-yearly meeting of the Governors was held in the recreation room of the nurses' new home, Sir Edward Wood, the chairman, presiding. He made an announcement which was received with the utmost cordiality and thankfulness that he had now succeeded in obtaining donations or promises for the full amount required by the reconstruction scheme. Roughly speaking, £100,000 had been raised and spent during the past eight years, and the Leicester Infirmary could now take a prominent place amongst hospitals, and its general equipment was considered to be as effective as its buildings. The great need now was to increase the income by another £3,000 per annum to provide for the additional cost of maintenance of the enlarged buildings and of the thirty-three beds now about to be opened, and he hoped now that the capital outlay was provided to be able to secure this. During the half-year 1,822 in-patients had been treated, the average number of beds daily occupied was 214.4, and 21,707 out-patients had been attended to, an increase under each heading. The total income was £8,135—all the items of income showing increases except legacies. Expenditure amounted to £10,520, an increase of £520, which the Board had outlined previously. Sir Edward Wood then describing several improvements desirable in the next few years (new floors and, if possible, balconies for the St. Matthew's block, etc.), said that it was not intended to ask for approval of an outlay on these improvements, for so much had been done recently that the income now must be developed; but the Board outlined them for the sake of any public benefactor in want of an opportunity and a scheme to carry through.

**Cottage Hospital, Felixstowe.**

Lady Beatrice Pretyman has opened recently the Cottage Hospital for Felixstowe and Walton, which the generosity of Mr. and Mrs. Croydon has given to the town. The foundation stone was laid a year ago on the site occupied previously by Mr. Croydon's house, and the money for the furnishing of the hospital was in hand, having been collected some time ago in case this satisfactory emergency should come to pass. Mr. E. G. Pretyman, M.P., the first President, received the deed of gift and title deeds of the building from Mr. Croydon, and alluded to the law's delay which might be expected from the voluntary dispositions duty under the new Finance Act, since it was not clear whether gifts for charitable purposes were exempt. Dr. Havell remarked on the opportunity which the new hospital would give to the medical men of the district, and appealed for its general support. Mr. Croydon hopes that the district or the District Council will provide an endowment fund, and the popular motive of raising a memorial to the late King will probably be appealed to for this purpose. The accommodation for patients comprises two wards, each with four beds, and two private wards. There is also a theatre with $x$-ray apparatus—a type of the up-to-date fittings aimed at throughout each department. Mr. Henry J. Wright, M.S.A., of Ipswich, is the architect.

**Swansea General and Eye Hospital.**

Established as a dispensary in 1814, as an infirmary in 1817, as a general hospital in 1867, and opened in 1878, the Swansea General and Eye Hospital does not propose to end its improvements there, and a comprehensive scheme of extensions is now being considered. This scheme is expected to cost £25,000, and the board of management hopes that the increase of work which the hospital is undertaking each year will be a proof to Swansea that these extensions are necessary. In-patients in the hospital's year just ended numbered 1,836, and the total number treated was 10,868 in all departments. The Convalescent Home received practically 280 of these to confirm their cure, and yet its income was only half its expenditure, the home thereby becoming a charge on the general fund. The ordinary income was £10,617, and the ordinary expenditure £9,815. Much attention has been given to the drainage system, and Mr. Lambert, the sanitary inspector to the Corporation, has supervised this work. Improvements are wanted in the pathological department in the form of a whole-time pathologist, partly because certain industrial diseases, for instance, nickel poisoning and pitch cancer, are found in Swansea and cannot be investigated fully as things are. The cost of maintenance is put at £500 a year, and it is pointed out that if the laboratory were thrown open to assist the medical men of South-west Wales part of this income would be spontaneously forthcoming.

**Brompton Hospital Sanatorium.**

Inspection day at the Frimley Sanatorium in connection with the Brompton Hospital for Consumption was held last Saturday, when visitors had an opportunity of observing the Frimley system, by which graduated work is given to patients, only first-stage or convalescent cases being received. Accommodation is provided for 150 patients, two-thirds of whom are men. The inspection was made all the more instructive by the presence of some 200 ex-patients, who showed what fine results this treatment of incipient phthisis has often achieved. Their present Majesties, when Prince and Princess of Wales, opened the Sanatorium in 1904. The inclusive cost per bed is £64 a year. It is satisfactory to note that those patients who were placed in the first grade were occupied with such genuine physical labour as the preparation of building foundations, on which it is hoped one day by their own efforts and other's subscriptions a chapel will rise. The women are often engaged in the garden, where they were trenching at the time of the inspection.

**Essex County Hospital.**

The most interesting thing about the half-yearly meeting of the Governors of the Essex County Hospital was, perhaps, the attempt made by one gentleman to make this the last half-yearly meeting ever held. Mr. Sparling moved that no half-yearly meeting of Governors be held, on the ground that its discontinuance would save expense and time. His motion, we would add, was defeated by one vote, and it is worth while to compare with the above reasoning the argument which Colonel Merriman quickly put forward on behalf of Governors' meetings as a whole. He said that one of the objects of such meetings as theirs was to advertise the hospital throughout the county, and in his opinion quarterly meetings might be even more useful for this purpose than the half-yearly ones they had now. We venture to think that the one vote which turned the scale in favour of Colonel Merriman's contention, or rather which prevented the existing scale from being upset, does not represent adequately the force of institutional opinion in favour of frequent periodic meetings of Governors. But he deserves to be thanked for emphasising the principle which underlies them. In order to show practically its force and value we make, on purpose, no mention this time of the facts and figures submitted to the meeting, leaving our readers to decide whether, by exciting public interest in such hospital questions as this, Governors' meetings, and Colonel Merriman with them, have not been justified beyond dispute.

**West Bromwich Hospital.**

The annual report of the West Bromwich District Hospital discloses the fact that while there has been a small and not surprising increase in the number of in-patients, there has been a complementary decrease in those attended at the out-patient department. The total number were in each case: for the in-patients 928, an increase of 32, and for the out-patients 9,539 in the general and 2,657 in the eye department, decreases of respectively 27 and 94. The subscriptions, however, have gone up and reached £742. It must not be supposed from this that the hospital is out of debt; far from it. A further £500 a year is being appealed for in order that, at the present rate of income and expenditure, the hospital may pay its way. The total amount of its debts is £2,667. It is curious, by the way, to note that the expenses during the year were less by twenty shillings than in the year ending July 1909. Owing to unfortunate ill-health among the nurses the hospital was obliged to spend more upon wages than it would otherwise have done.

